# Paclitaxel resistance in untransformed human mammary epithelial cells is associated with an aneuploidy-prone phenotype

**DOI:** 10.1038/sj.bjc.6603936

**Published:** 2007-10-30

**Authors:** B P Bouchet, J Bertholon, N Falette, C Audoynaud, C Lamblot, A Puisieux, C M Galmarini

**Affiliations:** 1 Université de Lyon, Lyon, F-69622, France; 2 Université Lyon 1, ISPB, Lyon, F-69003, France; 3 INSERM, U590, Lyon, F-69008, France; 4 IFR 62, Lyon, F-69008, France; 5 Centre LEON-BERARD, Oncologie Moléculaire, Lyon, F-69373, France; 6 Université Lyon 1, UFR de Médecine Lyon-Sud, Oullins, F-69921, France; 7 CNRS, UMR 5239, Oullins, F-69921, France; 8 IFR 128, Lyon, F-69365, France

**Keywords:** paclitaxel, drug resistance, aneuploidy, chromosomal instability, micronuclei, microtubule

## Abstract

Despite its increasing clinical use, almost no data are currently available about paclitaxel effects on non-cancerous mammary epithelial cells. We have previously established paclitaxel-resistant sub-cell lines (paclitaxel-surviving populations, PSPs; *n*=20), and sensitive controls (control clones, CCs; *n*=10), from the untransformed human mammary epithelial cell line HME1. In this study, we aimed to establish whether paclitaxel resistance was associated with a modified sensitivity to paclitaxel-induced aneuploidy. For this purpose, we analysed basal and paclitaxel-induced chromosome missegregation, apoptosis and aberrant spindle multipolarisation as well as microtubular network composition for each subline. PSP sublines showed higher basal and paclitaxel-induced chromosome missegregation than the CC sublines. This phenomenon was associated with resistance to paclitaxel-induced apoptosis. No significant difference in paclitaxel-induced spindle pole abnormalities between CC and PSP sublines was found. Besides, we showed that a majority of PSPs display a constitutively disrupted microtubular network composition due to aberrant tubulin expression and post-translational modifications. These results clearly indicate that paclitaxel resistance in untransformed human mammary epithelial cells is related to an increased susceptibility to acquire aneuploidy in response to this agent. The consequences of these paclitaxel-associated alterations could be deleterious as they can potentially trigger tumorigenesis.

Cytotoxicity is a desirable consequence of cancer chemotherapy. In most tumour cells, induction of genotoxic damage by anticancer agents favours cell death (Colella *et al*, 1999; Branch *et al*, 2000). However, increased genetic damages could also have adverse consequences if the affected cells are not malignant. In fact, it has been previously shown that genetic instability, characterised by an abnormal number of chromosomes, is associated with secondary malignancies. Thus, consideration of the potential aneugenicity of chemotherapy to humans is a necessary adjunct to its clinical use.

Paclitaxel is a chemotherapeutic agent that is frequently used in several human cancers, including lung, ovarian and breast cancer. Several previous works have addressed the aneugenic potential of this agent in various *in vitro* and *in vivo* models (Tinwell and Ashby, 1994; Jagetia and Adiga, 1995; Jagetia and Nayak, 1996; Digue *et al*, 1999; Galmarini *et al*, 2007). However, despite its increasing use, almost no data are currently available concerning its effects in normal human mammary epithelial cells. In a precedent work, we described that sub-cell lines of untransformed human mammary epithelial cells (HME1) were able to survive to a 1-week paclitaxel treatment (paclitaxel-surviving populations, PSPs) (Galmarini *et al*, 2006). In most of these sublines, the emergence of a transitory or stable drug resistance phenotype was related to the inactivation of p21/WAF1 protein.

In this study, we sought to determine whether paclitaxel resistance could be related to a modified sensitivity to paclitaxel aneugenicity. For this purpose, we firstly assayed whether paclitaxel treatment of PSPs and control clones (CCs) sublines induced alterations typically associated to aneuploidy appearance (chromosome missegregation and apoptosis defect). We secondly analysed the constitutive microtubular network composition and the aberrant spindle multipolarisation process after paclitaxel exposure in these sublines as potential causes for differential effect of paclitaxel on chromosomal stability. Our results show that paclitaxel resistance in untransformed mammary epithelial cells, characterised by an apoptosis defect, is associated with an aneuploidy-prone phenotype.

## MATERIALS AND METHODS

### Reagents

Paclitaxel was obtained from Bristol-Myers Squibb (Paris, France). Cytochalasin-B and Hoechst 33258 were purchased from Sigma (St Quentin Fallavier, France). Mouse fluorescein isothiocyanate (FITC)-conjugated anti-human *α*-tubulin, mouse anti-human *γ*-tubulin and rabbit tetramethylrhodamine isothiocyanate (TRITC)-conjugated anti-mouse IgG antibodies were purchased from DAKO (Glostrup, Denmark). Antibodies against *α*-tubulin (B-5-1-2), acetylated tubulin (6-11B-1), tyrosinated tubulin (Tub-1A2), *β*-tubulin (Tub 2.1) and *β*-actin (AC-15) were purchased from Sigma (St Quentin Fallavier, France); antibodies against class IV *β*-tubulin isotype were purchased from Biogenex (San Ramon, CA, USA). Class III *β*-tubulin isotype (TUJ1 clone) was kindly provided by Anthony Frankfurter (University of Virginia, Charlottesville, VA, USA).

### Obtention of PSPs

HME1 cells were purchased from ATCC collection (hTERT-HME1, telomerase-immortalised human mammary epithelial cells that stably express the telomerase catalytic subunit, hTERT). Paclitaxel-surviving populations were obtained from HME1 cells as reported previously (Galmarini *et al*, 2006). Briefly, HME1 cells were seeded onto 20 separate 96-well plates at a concentration of 100 cells/well. The following day, treatment with paclitaxel started at a dose of 10 pM over a period of 7 days. Paclitaxel-surviving Populations were allowed to grow for 2–3 weeks in paclitaxel-free culture medium. Wells containing PSPs were harvested, frozen and expanded in drug-free medium for experimental studies. As a control, 10 clones of HME1 cells (CCs) were obtained by limiting dilution (0.1 cell/well) with no exposure to the drug.

### Cytokinesis-block micronucleus assay

**Figure 1:**
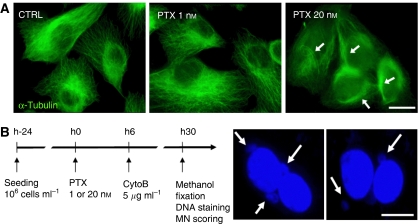
Effect of 1 and 20 nM paclitaxel on microtubular network and chromosome segregation in untransformed human mammary epithelial cells. (**A**) Immunostaining of *α*-tubulin (green) in untreated (CTRL), 1 and 20 nM paclitaxel-treated (PTX 1 and 20 nM) HME1 cells. White arrows indicate microtubule bundles. Scale bar, 10 *μ*m. (**B**) Micronucleus detection in paclitaxel-treated PSP and CC cells by CBMN assay. Briefly, 10^6^ cells ml^−1^ were seeded 24 h before paclitaxel (PTX) treatment (24 h). Cytochalasin-B (CytoB) at 5 *μ*g ml^−1^ was added in culture media 6 h after the beginning of paclitaxel exposure (6 h). After 30 h of paclitaxel treatment, cells were fixed by methanol, DNA was stained with Hoechst 33258 and binucleated cells with micronuclei (MN) were scored. White arrows indicate micronuclei in binucleated cytokinesis-blocked HME1 cells treated by 1 nM paclitaxel. Scale bar, 10 *μ*m. CBMN, cytokinesis-block micronucleus.

CC and PSP cells were seeded at a concentration of 10^6^ cells ml^−1^. Paclitaxel was added at two different concentrations (1 and 20 nM). After 6 h of paclitaxel treatment, cytochalasin-B (5 *μ*g ml^−1^) was added and cells were incubated at 37°C for 24 h and then fixed in 100% methanol. DNA was counterstained with Hoechst 33258 (5 *μ*g ml^−1^). Coverslips were examined with an Axioplan microscope (Carl Zeiss, Sartrouville, France) using a Zeiss × 100 1.3 oil-immersion objective. Pictures were acquired by using a confocal laser scanning TCS SP2 microscope (Leica, Le Pecq, France) and a × 63 oil-immersion objective. The main nucleus and micronuclei showed bright blue fluorescence (Figure 1B). The micronucleation index was determined for each subline by the use of two independent cytokinesis-block micronucleus (CBMN) assays. Each assay consisted in two micronuclei scoring in a minimum of 200 binucleated cells per experimental condition (total score on 400 cells). Criteria for scoring micronuclei in binucleated cells in the CBMN assay were described in detail by Fenech (2000). After 24 h of single cytochalasin-B treatment, the mean frequency of binucleated cells in CC and PSP were 55.1 and 60.1%, respectively.

### Western blots

Protein expression was determined by Western blot analysis in CC and PSP cells at basal conditions as described previously (Galmarini *et al*, 2001). Horizontal scanning was performed on Western blots by acquisition into Adobe Photoshop software (Apple, Cupertino, CA, USA).

### Apoptosis detection

Apoptotic index was calculated as the percentage of cells showing apoptotic morphology revealed by 5 *μ*g ml^−1^ Hoechst 33258 (Sigma, St Quentin Fallavier, France) staining of nuclei, after 72 h paclitaxel treatment (1 or 20 nM) or without treatment. For each experimental condition, a minimum of 200 cells per subline were analysed. Apoptosis was also evaluated in cytochalasin-B-treated cells, with or without 30 h paclitaxel treatment. For each independent CBMN assay, a minimum of 300 cells were analysed.

### Double-immunofluorescence staining

CC and PSP were grown on glass coverslips and treated for 24 h with paclitaxel at two different dose levels: 1 and 20 nM. To assay mitotic spindle pole impairments, cells were fixed with 4% paraformaldehyde and then permeabilised with 1% Triton X-100 in phosphate-buffered saline (PBS). After incubation with a blocking solution (5% bovine serum albumin (BSA) in PBS) for 30 min, cells were incubated with primary mouse anti-human *γ*-tubulin antibody diluted 1/1000 for 1 h at 37°C in a humid chamber. After three washes with a PBS/BSA1% solution, cells were incubated for 30 min with a TRITC-conjugated goat anti-mouse IgG secondary antibody. Then cells were incubated with a FITC-conjugated mouse anti-human-*α*-tubulin diluted 1/100. Finally, DNA was counterstained by the addition of Hoechst 33258 (1 *μ*g ml^−1^) for 5 min. Cells were observed with a Zeiss Axioplan microscope using a Zeiss × 100 1.3 oil-immersion objective. The spindle pole abnormalities were scored in a minimum of 300 mitotic cells per subline and for each condition (baseline, 1 and 20 nM paclitaxel).

### Time-lapse imaging

We established HME1 cell line expressing histone H2B-GFP (H2B-GFP) by stably transfecting hTERT-HME1 cells with plasmid pBOS-H2BGFP (BD Biosciences, Erembodegem, Belgium) under blasticidin selection (5 *μ*g ml^−1^). Exponentially growing cells were plated on six-well culture plate and incubated with paclitaxel (1 nM for 24 h). Images were acquired on a Zeiss Axiovert photomicroscope with a PowerShot G5 Digital Camera (Canon, Courbevoie, France) under 37°C heated atmosphere. Together with the fluorescent images, transmitted light (differential interference contrast optics) images were acquired. Data were analysed using the Image J software.

### Statistical analysis

The differences between groups were tested by the Mann–Whitney *U* non-parametric test using the statistical package SPSS V10™. The level of significance used in all the analyses was *P*<0.05.

## RESULTS

### Paclitaxel induces persistent chromosome missegregation in PSP but not in CC sublines

To evaluate the chromosome segregation process after paclitaxel treatment in CC and PSP sublines, we performed CBMN assays. These assays were carried out without the use of specific markers of chromosomes, and thus estimated the global missegregation process but did not specify associated chromosome aberrations (for example, nucleoplasmic bridges, chromosome non-disjunction and breakage). We decided to test effects of 1 and 20 nM paclitaxel on chromosome segregation, as these two doses were previously found to be the paclitaxel IC_20_ and IC_80_ in HME1 cell line (Galmarini *et al*, 2006). Moreover, these doses act differentially to induce cell death in untransformed mammary cells. As shown in Figure 1A, 1 nM paclitaxel did not cause massive disorganisation of microtubule network, whereas 20 nM paclitaxel induced microtubule bundles in HME1 cells.

At baseline conditions, none of the CCs presented binucleated cells with micronuclei (Figure 1B; Table 1). In contrast, PSPs showed basal micronucleation (mean frequency of binucleated cells with micronuclei=4.9%). This increased frequency was due to four PSPs (PSP2, PSP4, PSP6 and PSP21; ranging from 16.7 to 40%) showing high basal micronucleation (Supplementary Table 1). Hence, some PSP sublines present constitutive chromosome aberrations compared with the sensitive CC sublines.

At dose of paclitaxel 1 nM, 60% (6/10) of CCs showed micronucleation in binucleated cells (Supplementary Table 1) with a mean frequency of 1.1% (ranging from 0 to 4.1%). Paclitaxel at the same dose also increased the frequency of micronucleation in PSPs. After this treatment, 80% of PSPs (16/20) showed binucleated cells with micronuclei; the mean frequency of micronucleation was 4.9% (range: 0–14.6%) (Table 1 and Supplementary Table 1). Strikingly, the four PSPs that shared a high basal micronucleation (PSP2, PSP4, PSP6 and PSP21) showed a decreased micronucleation after 1 nM paclitaxel exposure.

After 20 nM paclitaxel exposure, massive cell death induced in CC sublines during the CBMN assay did not permit to score micronucleation in binucleated cells (Table 1, Supplementary Table 1). In contrast, at the same time point, 80% of PSP (16/20) sublines presented binucleated cells with micronuclei after drug treatment (Supplementary Table 1). Besides, the mean frequency of micronucleation in PSP binucleated cells was 42.5% (Table 1).

### PSP sublines are resistant to paclitaxel-induced apoptosis

We sought to determine if the increased paclitaxel-induced chromosome aberrations observed in PSPs was associated with a defect of paclitaxel-induced cell death. For this purpose, we treated all sublines with this agent at doses of 1 and 20 nM, for 72 h, and we evaluated the apoptotic index (Table 1). In CC cells, paclitaxel treatment induced apoptosis in a dose-dependent manner (1 nM: 3.5%; 20 nM: 62.6%). Similarly, PSPs showed a dose-dependent paclitaxel-induced apoptosis; however, apoptotic index was significantly lower in PSP than in CC sublines (1 nM: 1.2%, *P*<0.05; 20 nM: 34.5%, *P*<0.001).

To establish a correlation between cell death and micronucleation in CCs and PSPs, we counted apoptosis in those slides used for micronucleation counting by CBMN assay. Notably, a similar significant apoptotic tendency than that observed after 72 h was already observed in PSP compared with CC sublines after 30 h of combined 20 nM paclitaxel-cytochalasin-B treatment (CC: 45.1%, PSP: 35.4%, *P*<0.05; Supplementary Figure 1). This result was corroborated by the observation of a significantly lower binucleation in CC compared with PSP sublines, after 20 nM paclitaxel treatment, indicative of an increased cell death and/or cell-cycle delay in these sublines (CC: 1.0%, PSP: 6.0%, *P*<0.01; Supplementary Table 2). When performing a more detailed analysis, we observed that ‘unstable’ PSP sublines (U-PSP), namely those that showed constitutive chromosome segregation defect (PSP2, PSP4, PSP6 and PSP21; Table 1), exhibited significantly more apoptosis than stable PSP sublines (S-PSP) when analysed after combined 1 nM paclitaxel-cytochalasin-B treatment (U-PSP: 13.2%, S-PSP: 4.1%, *P*<0.05; Supplementary Figure 1).

### PSP sublines display constitutive disruptions of the microtubular network composition

**Figure 2:**
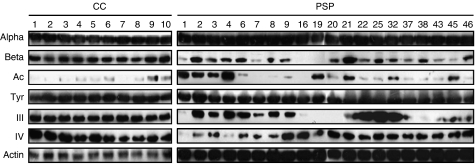
Microtubular network composition in CC and PSP sublines. Expression of total *α*-tubulin (*Alpha*), *β*-tubulin (*Beta*), acetylated *α*-tubulin (*Ac*), tyrosinated *α*-tubulin (*Tyr*), class III *β*-tubulin (*III*), class IV *β*-tubulin (*IV*) and *β*-actin (*Actin*) were analysed by Western blot in whole-cell lysates of exponentially growing cells from CC (1–10) and PSP (1–46) sublines. CC, control clone; PSP, paclitaxel-surviving population.

Numerous authors suggested that alterations of microtubule dynamics could lead to chromosome missegregation and aneuploidy (ter Haar *et al*, 1996; Sorger *et al*, 1997; Rosa *et al*, 2006). Moreover, it is well established that composition of microtubule network – particularly tubulin isotype expression and tubulin post-translational modifications – closely influences microtubule dynamics and resistance to paclitaxel (Ohta *et al*, 1994; Kavallaris *et al*, 1997; Ranganathan *et al*, 1998; Carles *et al*, 1999; Banerjee, 2002). For this reason, we decided to investigate the microtubular network composition in PSP compared with CC sublines. We thus analysed the expression of total *α*- and total *β*-tubulin, acetylated- and tyrosinated-*α*-tubulin, and class III and class IV *β*-tubulin. In the CC, total *α*- and total *β*-tubulin were uniformly expressed (Figure 2). The levels of protein expression of acetylated *α*-tubulin and tyrosinated *α*-tubulin were unchanged between the different CCs; however, the levels of protein expression of acetylated *α*-tubulin were low compared with those observed for tyrosinated *α*-tubulin. Class III and class IV *β*-tubulins were similarly expressed in the CCs.

In the PSPs, total *α*-tubulin expression was expressed in similar levels in all the sublines. However, total *β*-tubulin protein content was highly altered in several PSP sublines including PSP1, PSP7, PSP16, PSP19, PSP43 and PSP45 (Figure 2). Indeed, these sublines presented a decreased expression of total *β*-tubulin compared with CCs and other PSPs. When analysing the expression of acetylated *α*-tubulin, we observed that protein levels were different between the different sublines, with higher contents of acetylated tubulin protein in PSP1, PSP2, PSP3, PSP4, PSP19, PSP21, PSP32 and PSP45. In these PSPs, compared with the CCs, the acetylated *α*-tubulin was found to be overexpressed. These variations in protein expression were not observed for tyrosinated *α*-tubulin. Compared with CCs, the amounts of class III *β*-tubulin were greatly decreased in PSP1, PSP16, PSP19, PSP20, PSP38 and PSP43. In contrast PSP2, PSP4, PSP22, PSP25 and PSP32 presented higher levels of class III *β*-tubulin than those observed in CC sublines. Finally, the amounts of class IV *β*-tubulin showed to be highly decreased in PSP1, PSP2 and PSP4. These data indicate that the majority of PSP sublines exhibit a disrupted microtubular network when compared with sensitive sublines.

### PSP and CC sublines undergo dose-dependent aberrant spindle multipolarisation after paclitaxel treatment

**Figure 3:**
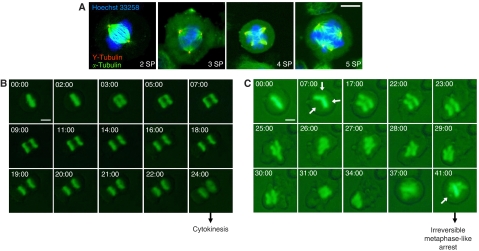
Spindle pole aberrations in untransformed human mammary epithelial cells after paclitaxel treatment. (**A**) Double-immunofluorescence staining was performed to detect *γ*-tubulin (red) and *α*-tubulin (green). DNA was counterstained with Hoechst 33258 (blue). Panels show representative mitotic figures of CC cells with two (2SP), three (3SP), four (4SP) or five (5SP) spindle poles. Scale bar, 10 *μ*m. (**B**) Representative time-lapse imaging of chromosomes in an untreated H2B-GFP expressing HME1 cell during metaphase–telophase transition. As indicated, cytokinesis was completed 24 min after metaphase (24:00). (**C**) Representative time-lapse imaging of chromosomes in a 1 nM paclitaxel-treated H2B-GFP expressing HME1 cell during metaphase–telophase transition. White arrows on 07:00 panel indicate the tripolar spindle orientation. Of note, 41 min (41:00) after metaphase, cell failed to enter telophase and irreversibly arrested in a metaphase-like arrest showing unaligned chromosome (white arrow). Time, mm : ss normalised to metaphase equals 00 : 00. Scale bar, 10 *μ*m.

We aimed to verify whether chromosome missegregation process observed in PSPs could be associated to aberrant mitotic spindle polarisation. For this purpose, we evaluated the spindle pole status by double-immunofluorescence staining of *α*-tubulin and *γ*-tubulin in untreated and paclitaxel-treated (1 and 20 nM) PSP sublines compared with CC sublines (Figure 3A, Table 1). Percentage of normal bipolar and multipolar mitoses was determined as described in Material and Methods.

At baseline, the vast majority of mitoses in CC sublines were normal, with almost all cells showing a bipolar spindle (Table 1). When treated with paclitaxel, the CC cells revealed a dose-dependent increase of mitotic multipolarisation (1 nM: 13.9%; 20 nM: 73.2%). Similarly, at baseline, most of PSPs had normal mitoses with 95.1% of cells showing a bipolar spindle (Table 1). When treated with paclitaxel, PSPs displayed a mean percentage of multipolar mitoses of 7.3 and 85.2% after 1 and 20 nM paclitaxel exposure, respectively. In this sublines group, the percentage of mitoses with more than two poles also increased in a dose-dependent manner (Table 1). Notably, compared with CCs, PSPs seemed to show less mitotic multipolarisation after 1 nM paclitaxel treatment; in contrast, PSP sublines seemed to show more mitotic multipolarisation after 20 nM; however, these differences were not statistically significant (*P*>0.05).

To confirm that the previously observed paclitaxel-associated spindle multipolarisation in fixed samples were not due to methodological artifacts, we evaluated the real-time effects of paclitaxel in the HME1 cell line stably expressing a histone H2B-green fluorescent protein (GFP). Expression of this fusion protein allows sensitive analysis of chromosome dynamics as these structures are fluorescently labelled in living cells (Kanda *et al*, 1998). The cell divisions observed in the control situation (Figure 3B) were highly comparable with our observations in fixed samples. This implies that the intensity of the lamp beam did not cause significant cell-cycle arrest and predominantly allowed mitoses to occur normally.

Mitotic progression was analysed on real time in 1 nM paclitaxel-treated cells (*n*=10) compared with untreated cells (*n*=10) (Figure 3B–C). Untreated cells generally, completed metaphase–telophase progression in approximately 25 min (Figure 3B). In contrast, most of the 1 nM paclitaxel-treated cells showed delayed mitosis and aborted telophase, especially in cells in which appearance of multipolar spindle was observed (Figure 3C). After paclitaxel exposure, most of the recorded multipolarised mitoses led to initial metaphase arrest or aborted telophase followed by irreversible metaphase-like arrest characterised by unaligned chromosomes (Figure 3C and data not shown).

## DISCUSSION

In a precedent work, we have shown that paclitaxel-resistant sublines (PSPs) can be established from an untransformed human mammary epithelial cell line (Galmarini *et al*, 2006). In this study, we aimed to further investigate the response of these drug-resistant untransformed cells to paclitaxel. For this purpose, we evaluated the effects of this agent on mitotic chromosome segregation, as well as apoptotic response of PSP sublines compared with control-sensitive sublines (CCs). Our results clearly indicate that paclitaxel-resistant untransformed human mammary epithelial cells have an increased susceptibility to develop aneuploidy in response to this agent. Our data also suggest that disruption of microtubular network, a well-known paclitaxel-resistant mechanism, could contribute to this aneuploidy-prone phenotype.

Our *in vitro* experiments showed that after paclitaxel treatment, PSP sublines presented more paclitaxel-induced chromosome missegregation than paclitaxel-sensitive sublines. Furthermore, our results suggest that the lower level of micronucleation observed in paclitaxel-treated CC sublines could be explained by a rapid cell death onset that eliminates CC cells displaying dose-dependent chromosome aberrations. Given the fact that this would be consistent with their sensitive status, further kinetic investigation of apoptotic response to paclitaxel in these sublines will be needed to ascertain this issue (Galmarini *et al*, 2006). Additionally, PSPs also showed lower paclitaxel-induced apoptotic levels, consistent with their previously described resistant status (Galmarini *et al*, 2006). The combination of apoptosis defect with chromosome missegregation have been extensively described as a driving force responsible for aneuploidy in tumour cells (Hollander *et al*, 1999; Jallepalli and Lengauer, 2001; Rajagopalan and Lengauer, 2004; Weaver *et al*, 2007). Thus, our results strongly suggest that paclitaxel-resistant untransformed mammary cells are characterised by an aneuploidy-prone response to paclitaxel treatment. To our knowledge, this is the first study identifying the increased susceptibility to aneuploidisation in untransformed human mammary epithelial cells as an advantage under paclitaxel treatment.

Of note, four PSP sublines (PSP2, PSP4, PSP6 and PSP21) showed a substantially increased level of constitutive chromosome missegregation as shown by an increased level of micronuclei at baseline. Strikingly, these four PSPs presented a diminished level of chromosome missegregation after 1 nM paclitaxel treatment. Moreover, we found that these ‘unstable’ PSPs underwent significantly more apoptosis than ‘stable’ PSP sublines after short-term 1 nM paclitaxel treatment (CBMN assays, 30 h). All these results suggest a rapid elimination of cells in response to overmassive chromosome aberrations. Our data would be in accordance with recent works that indicate that high level of aneuploidy leads to cell lethality and/or could avoid stable selection of phenotype with growth advantage (Weaver *et al*, 2007). However, additional investigations will be necessary to specify cell death response in paclitaxel-resistant untransformed mammary cells.

Our data also revealed that aneuploidy-prone phenotype in untransformed mammary cells could be, at least in part, driven by alterations in the composition of microtubular network. Indeed, the majority of PSPs presented constitutive altered levels of total *β*-tubulin protein content, acetylated *α*-tubulin and/or class III *β*-tubulin. The link between aberrant modifications of microtubule dynamics and paclitaxel resistance was yet established in paclitaxel-resistant cells through identification of alterations in tubulin isotype expression and/or post-translational modifications. Indeed, several groups have described association of increased expression of total *β*-tubulin, class III *β*-tubulin and/or acetylated *α*-tubulin with paclitaxel resistance (Ohta *et al*, 1994; Kavallaris *et al*, 1997; Ranganathan *et al*, 1998; Carles *et al*, 1999; Banerjee, 2002). Furthermore, several studies have demonstrated that microtubule defects, such as changes in the expression of tubulins or mutations in the tubulins genes, can lead to chromosome missegregation (ter Haar *et al*, 1996; Sorger *et al*, 1997; Asakawa *et al*, 2006; Rosa *et al*, 2006). Thus, it is conceivable that cells that exhibit paclitaxel resistance related to aberrant tubulin expression would also show an increased susceptibility to chromosome missegregation, as that observed in PSP sublines. Notably, the diversity of tubulin alterations in PSP sublines did not permit to identify specific tubulin patterns inducing drug resistance, but rather an association between globally imbalanced tubulin ratios and paclitaxel-induced chromosome missegregation susceptibility. Additionally, it remains unclear how these different modified tubulin ratios affect the stability of microtubules in PSPs and their sensitivity to paclitaxel. However, our results indicate that, in paclitaxel-resistant untransformed human mammary epithelial cells, association of microtubular network disruption with apoptosis defect could greatly contribute to trigger aneuploidy in response to paclitaxel. As various processes could associate microtubule defects and paclitaxel resistance, further studies should ascertain mechanisms that impede microtubule-related cell death initiation in PSP sublines (Davis and Johnson, 1999; Nuydens *et al*, 2000; Mollinedo and Gajate, 2003). Of note, an other interesting issue would consist in determining whether modified tubulin expressions have been initially selected in HME1 cells because of their paclitaxel-resistance properties or whether they result from paclitaxel-induced aberrant but viable phenotypes.

Finally, even if our data show a dose-dependent increase of spindle multipolarisation, associated with aberrant chromosomes behaviour in both PSP and CC sublines after paclitaxel treatment, no significant difference concerning this process was found between the two sublines group. We cannot exclude that differences could be detected at different post-paclitaxel treatment times of analysis. Hence, further kinetic studies will be necessary to specify the response to paclitaxel in regard to spindle pole abnormalities in PSP sublines.

Recent works claimed that high rates of drug resistance in cancer cells are caused by aneuploidy-catalysed generation of resistance-specific aneusomies (Li *et al*, 2005; Duesberg *et al*, 2007). Hence, we speculate that plasticity of chromosomal patrimony in PSPs could be linked to their ability to escape from paclitaxel-induced apoptosis. Indeed, our results show that these sublines are prone to acquire karyotype modifications. The resulting massive gene dosage modifications could explain their predisposition to acquire paclitaxel resistance. In contrast, cells that retain chromosomal stability, unable to adapt to paclitaxel-induced chromosome aberrations, would undergo efficient apoptosis. Other previous works prompt us to envisage that paclitaxel doses that could trigger moderate aneuploidy could also efficiently promote the acquisition of a resistant phenotype (Weaver *et al*, 2007). Thus, further investigations should aim to precise interrelations between drug-resistance appearance and aneuploidisation in untransformed cells.

In summary, our results strongly indicate that paclitaxel resistance in untransformed human mammary epithelial cells is associated with the emergence of chromosome missegregation and disrupted microtubular network. In addition, these non-cancerous cells are deficient for paclitaxel-induced apoptosis and thus, characterised by an aneuploidy-prone phenotype related to paclitaxel treatment. The consequences of these paclitaxel effects in non-tumour cells could be deleterious as the potentially resulting chromosomal instability could promote tumorigenesis hence the appearance of a secondary neoplasm linked to paclitaxel treatment.

## Figures and Tables

**Table 1:** Micronucleation, apoptosis and spindle pole status in CC and PSP sublines after paclitaxel treatment

	**MN-BN^a^**			**Apoptosis^b^**			**Spindle pole status^c^**					
							**Control**		**Paclitaxel 1 nM**		**Paclitaxel 20 nM**	
	**Control**	**Paclitaxel 1 nM**	**Paclitaxel 20 nM**	**Control**	**Paclitaxel 1 nM**	**Paclitaxel 20 nM**	**SP=2**	**SP>2**	**SP=2**	**SP>2**	**SP=2**	**SP>2**
CC	0.0	1.1	ND	0.2	3.5	62.6	95.5	4.5	86.0	13.9	26.8	73.2
PSP	4.9	4.9^*^	42.5	0.5	1.2^*^	34.5^***^	95.1	4.9	92.7	7.3	14.8	85.2

PSP, paclitaxel-surviving population.

a Percentage of binucleated cells showing micronucleation; values represent mean percentage of micronucleated cells in CC (*n*=10) and PSP subline (*n*=20) groups.

b Percentage of cells showing apoptotic morphology; values represent mean percentage of apoptotic cells in CC (*n*=10) and PSP subline (*n*=20) groups.

c Percentage of mitotic cells with 2 (SP=2) or more spindle poles (SP>2); values represent mean percentage of each category in CC (*n*=10) and PSP subline (*n*=20) groups.

^*^P<0.05, ^***^P<0.001, comparison between CC and PSP values. ND, not determined.
