# Supplementary material for: Paclitaxel resistance in untransformed human mammary epithelial cells is associated with an aneuploidy-prone phenotype
Source: Br J Cancer. 2007 Oct 30;97(9):1218–24. doi: 10.1038/sj.bjc.6603936 (PMC2360475; doi:10.1038/sj.bjc.6603936)
Supplement: Supplementary Table 1 and Table 2 [file 6603936x1.doc]

**Supplementary Table 1.** Micronucleation, apoptosis and spindle poles status in CC and PSP sublines after paclitaxel treatment.

|  | MN-BN*a* | | | | |  | Apoptosis*b* | | | | |  | Spindle Poles Status*c* | | | | | | | |
| --- | --- | --- | --- | --- | --- | --- | --- | --- | --- | --- | --- | --- | --- | --- | --- | --- | --- | --- | --- | --- |
|  | Control | Paclitaxel 1 nM | | Paclitaxel 20 nM | |  | Control | Paclitaxel 1 nM | | Paclitaxel 20 nM | |  | Control | |  | Paclitaxel 1 nM | |  | Paclitaxel 20 nM | |
|  |  |  |  |  |  |  |  |  |  |  |  |  | SP=2 | SP>2 |  | SP=2 | SP>2 |  | SP=2 | SP>2 |
| CC1 | 0.0 | 1.6 | 🡕*d* | ND |  |  | 0.5 | 5.5 | 🡕 | 60.4 | 🡕 |  | 96.4 | 3.6 |  | 95.8 | 4.2 |  | 64.3 | 35.7 |
| CC2 | 0.0 | 0.0 | - | ND |  |  | 0.0 | 6.0 | 🡕 | 57.0 | 🡕 |  | 95.5 | 4.5 |  | 90.0 | 10.0 |  | 50.0 | 50.0 |
| CC3 | 0.0 | 1.6 | 🡕 | ND |  |  | 1.0 | 2.0 | 🡕 | 60.0 | 🡕 |  | 96.1 | 3.9 |  | 92.5 | 7.1 |  | 1.5 | 98.5 |
| CC4 | 0.0 | 4.1 | 🡕 | ND |  |  | 0.0 | 8.0 | 🡕 | 64.0 | 🡕 |  | 93.8 | 6.2 |  | 62.9 | 37.1 |  | 1.6 | 98.4 |
| CC5 | 0.0 | 1.3 | 🡕 | ND |  |  | 0.0 | 1.8 | 🡕 | 69.7 | 🡕 |  | 90.0 | 10.0 |  | 77.8 | 22.2 |  | 63.3 | 36.7 |
| CC6 | 0.0 | 1.1 | 🡕 | ND |  |  | 0.0 | 0.4 | 🡕 | 64.9 | 🡕 |  | 96.8 | 3.2 |  | 84.3 | 15.7 |  | 2.0 | 98.0 |
| CC7 | 0.0 | 0.0 | - | ND |  |  | 0.0 | 7.5 | 🡕 | 47.3 | 🡕 |  | 98.6 | 1.4 |  | 97.9 | 2.1 |  | 32.1 | 67.9 |
| CC8 | 0.0 | 0.0 | - | ND |  |  | 0.0 | 1.8 | 🡕 | 65.4 | 🡕 |  | 95.4 | 4.6 |  | 73.9 | 26.1 |  | 1.8 | 98.2 |
| CC9 | 0.0 | 0.0 | - | ND |  |  | 0.0 | 1.8 | 🡕 | 64.0 | 🡕 |  | 95.2 | 4.8 |  | 93.9 | 6.3 |  | 27.2 | 72.8 |
| CC10 | 0.0 | 1.7 | 🡕 | ND |  |  | 0.0 | 0.5 | 🡕 | 73.1 | 🡕 |  | 97.5 | 2.5 |  | 91.2 | 8.8 |  | 23.8 | 76.2 |
| PSP1 | 0.0 | 3.9 | 🡕 | 21.4 | 🡕 *e* |  | 0.5 | 0.0 | 🡖 | 31.7 | 🡕 |  | 97.8 | 2.2 |  | 96.0 | 4.0 |  | 5.0 | 95.0 |
| PSP2 | 40.0 | 1.0 | 🡖 | 11.8 | 🡕 |  | 1.5 | 1.9 | 🡕 | 39.1 | 🡕 |  | 93.7 | 6.3 |  | 91.4 | 8.6 |  | 16.7 | 83.3 |
| PSP3 | 0.0 | 0.8 | 🡕 | 22.2 | 🡕 |  | 0.0 | 8.6 | 🡕 | 49.3 | 🡕 |  | 95.0 | 5.0 |  | 76.1 | 23.9 |  | 4.8 | 95.2 |
| PSP4 | 16.7 | 9.9 | 🡖 | 28.6 | 🡕 |  | 1.0 | 0.5 | 🡖 | 27.1 | 🡕 |  | 97.0 | 3.0 |  | 94.9 | 5.1 |  | 21.6 | 78.4 |
| PSP6 | 16.7 | 10.8 | 🡖 | 35.0 | 🡕 |  | 0.5 | 2.7 | 🡕 | 49.8 | 🡕 |  | 94.4 | 5.6 |  | 84.6 | 15.4 |  | 7.6 | 92.4 |
| PSP7 | 0.0 | 2.0 | 🡕 | 50.0 | 🡕 |  | 0.0 | 0.5 | 🡕 | 52.4 | 🡕 |  | 99.0 | 1.0 |  | 97.9 | 2.1 |  | 2.6 | 97.4 |
| PSP8 | 0.0 | 9.5 | 🡕 | 100.0 | 🡕 |  | 0.0 | 0.0 | - | 26.8 | 🡕 |  | 98.6 | 1.4 |  | 97.9 | 2.1 |  | 0.0 | 100.0 |
| PSP9 | 0.0 | 13.4 | 🡕 | 60.0 | 🡕 |  | 0.0 | 0.0 | - | 47.3 | 🡕 |  | 95.9 | 4.1 |  | 100.0 | 0.0 |  | 32.8 | 67.2 |
| PSP16 | 0.0 | 0.0 | - | 50.0 | 🡕 |  | 0.0 | 0.0 | - | 32.7 | 🡕 |  | 95.4 | 4.6 |  | 90.1 | 9.9 |  | 7.3 | 92.7 |
| PSP19 | 0.0 | 0.0 | - | 42.9 | 🡕 |  | 0.0 | 1.9 | 🡕 | 29.7 | 🡕 |  | 91.6 | 8.4 |  | 96.1 | 3.9 |  | 12.0 | 88.0 |
| PSP20 | 0.0 | 5.9 | 🡕 | 75.0 | 🡕 |  | 0.0 | 0.0 | - | 24.8 | 🡕 |  | 93.1 | 5.2 |  | 91.3 | 8.7 |  | 37.2 | 62.8 |
| PSP21 | 25.0 | 4.4 | 🡖 | 20.0 | 🡕 |  | 1.0 | 0.0 | 🡖 | 33.8 | 🡕 |  | 92.0 | 8.0 |  | 92.7 | 7.3 |  | 17.4 | 82.6 |
| PSP22 | 0.0 | 14.6 | 🡕 | 0.0 | 🡖 |  | 0.0 | 0.5 | 🡕 | 50.5 | 🡕 |  | 95.5 | 4.5 |  | 96.2 | 3.8 |  | 36.9 | 63.1 |
| PSP25 | 0.0 | 4.6 | 🡕 | 100.0 | 🡕 |  | 0.0 | 2.0 | 🡕 | 27.5 | 🡕 |  | 92.1 | 7.9 |  | 90.5 | 9.5 |  | 11.1 | 88.9 |
| PSP32 | 0.0 | 9.8 | 🡕 | 100.0 | 🡕 |  | 0.0 | 0.0 | - | 35.8 | 🡕 |  | 97.7 | 2.3 |  | 98.5 | 1.5 |  | 6.7 | 93.3 |
| PSP37 | 0.0 | 0.0 | - | 33.3 | 🡕 |  | 0.0 | 2.5 | 🡕 | 24.8 | 🡕 |  | 96.0 | 4.0 |  | 94.0 | 6.0 |  | 33.3 | 66.7 |
| PSP38 | 0.0 | 2.0 | 🡕 | 100.0 | 🡕 |  | 2.0 | 0.0 | 🡖 | 12.8 | 🡕 |  | 96.0 | 4.0 |  | 85.9 | 14.1 |  | 17.2 | 82.8 |
| PSP43 | 0.0 | 0.0 | - | 0.0 | - |  | 1.0 | 2.2 | 🡕 | 25.9 | 🡕 |  | 92.4 | 7.6 |  | 93.0 | 7.0 |  | 4.2 | 95.8 |
| PSP45 | 0.0 | 3.0 | 🡕 | 0.0 | 🡖 |  | 1.0 | 0.0 | 🡖 | 43.8 | 🡕 |  | 88.7 | 11.3 |  | 89.9 | 10.1 |  | 2.7 | 97.3 |
| PSP46 | 0.0 | 2.6 | 🡕 | 0.0 | 🡖 |  | 1.0 | 0.0 | 🡖 | 24.4 | 🡕 |  | 99.3 | 0.7 |  | 97.7 | 2.3 |  | 18.3 | 81.7 |

*a* percentage of binucleated cells showing micronucleation in CC and PSP sublines.

*b* percentage of cells showing apoptotic morphology in CC and PSP sublines.

*c* percentage of mitotic cells with 2 (SP=2) or more spindle poles (SP>2) in CC and PSP sublines.

*d* arrows in “Paclitaxel 1 nM” columns means increased (🡕), decreased (🡖) or unchanged (-) value in 1 nM paclitaxel treated sublines compared with untreated sublines.

*e* arrows in “Paclitaxel 20 nM” columns means increased (🡕), decreased (🡖) or unchanged (-) value in 20 nM paclitaxel treated sublines compared with 1 nM paclitaxel treated sublines.

ND, not determined.

**Supplementary Table 2.** Frequency of binucleated cells in cytochalasin-B treated CC and PSP sublines with or without paclitaxel treatment.

|  | BN*a* | | | | |
| --- | --- | --- | --- | --- | --- |
|  | Control |  | Paclitaxel 1 nM |  | Paclitaxel 20 nM |
|  | + |  | + |  | + |
|  | CytoB |  | CytoB |  | CytoB |
| CC1 | 64.2 |  | 62.3 |  | 0.5 |
| CC2 | 46.5 |  | 29.7 |  | 1.5 |
| CC3 | 44.2 |  | 29.0 |  | 1.4 |
| CC4 | 52.6 |  | 35.4 |  | 0.5 |
| CC5 | 62.2 |  | 37.3 |  | 0.0 |
| CC6 | 33.3 |  | 42.9 |  | 0.5 |
| CC7 | 57.4 |  | 15.8 |  | 0.5 |
| CC8 | 49.5 |  | 37.6 |  | 3.0 |
| CC9 | 58.0 |  | 62.4 |  | 0.5 |
| CC10 | 75.6 |  | 54.9 |  | 0.5 |
| **CC-Mean***b* | **54.4** |  | **40.7** |  | **1.0** |
| PSP1 | 79.2 |  | 87.9 |  | 7.0 |
| PSP2 | 60.3 |  | 50.7 |  | 8.5 |
| PSP3 | 64.0 |  | 59.7 |  | 8.8 |
| PSP4 | 49.4 |  | 59.5 |  | 10.4 |
| PSP6 | 66.7 |  | 44.8 |  | 9.6 |
| PSP7 | 67.8 |  | 48.3 |  | 3.0 |
| PSP8 | 66.8 |  | 36.8 |  | 3.5 |
| PSP9 | 53.7 |  | 32.1 |  | 2.5 |
| PSP16 | 55.9 |  | 43.7 |  | 4.0 |
| PSP19 | 64.2 |  | 55.3 |  | 9.9 |
| PSP20 | 71.0 |  | 54.4 |  | 7.7 |
| PSP21 | 45.6 |  | 22.0 |  | 7.4 |
| PSP22 | 60.1 |  | 23.9 |  | 3.8 |
| PSP25 | 71.6 |  | 59.8 |  | 1.0 |
| PSP32 | 69.7 |  | 53.1 |  | 1.5 |
| PSP37 | 65.7 |  | 32.4 |  | 4.4 |
| PSP38 | 62.4 |  | 45.7 |  | 1.0 |
| PSP43 | 60.4 |  | 46.8 |  | 0.5 |
| PSP45 | 58.2 |  | 32.4 |  | 24.8 |
| PSP46 | 69.6 |  | 53.7 |  | 0.5 |
| **PSP-Mean***c* | **63.1** |  | **47.1** |  | **6.0**** |

*a* percentage of binucleated cells in CC and PSP sublines.

*b* mean percentage of binucleated cells in CC sublines group.

*c* mean percentage of binucleated cells in PSP sublines group.

CytoB: cytochalasin-B

***P*<0.01, comparison between CC-Mean and PSP-Mean values.

**Figures**

**Supplementary Figure 1. Apoptosis in cytochalasin-B treated CC and PSP sublines with or without paclitaxel. A.** Percentage of apoptosis (% Apoptosis) in untreated (CTRL), 1 nM or 20 nM paclitaxel (PTX 1 nM, PTX 20 nM) treated CC (n=10) or PSP (n=20) sublines submitted to CBMN assay. **B.** Percentage of apoptosis (% Apoptosis) in "stable" (S-PSP: PSP1, PSP3, PSP7, PSP8, PSP9, PSP16, PSP19, PSP20, PSP22, PSP25, PSP32, PSP37, PSP38, PSP43, PSP45, PSP46. n=16) and "unstable" (U-PSP: PSP2, PSP4, PSP6, PSP21. n=4) PSP sublines submitted to CBMN assay after 1 nM paclitaxel treatment. *P* value determined by Mann-Whitney U non-parametric test; A: comparison between CC and PSP values, B: comparison between S-PSP and U-PSP values. NS: non significant.
